# Holt Oram syndrome: a registry-based study in Europe

**DOI:** 10.1186/s13023-014-0156-y

**Published:** 2014-10-25

**Authors:** Ingeborg Barisic, Ljubica Boban, Ruth Greenlees, Ester Garne, Diana Wellesley, Elisa Calzolari, Marie-Claude Addor, Larraitz Arriola, Jorieke EH Bergman, Paula Braz, Judith LS Budd, Miriam Gatt, Martin Haeusler, Babak Khoshnood, Kari Klungsoyr, Bob McDonnell, Vera Nelen, Anna Pierini, Annette Queisser-Wahrendorf, Judith Rankin, Anke Rissmann, Catherine Rounding, David Tucker, Christine Verellen-Dumoulin, Helen Dolk

**Affiliations:** Zagreb Children’s Hospital, Medical School, University of Zagreb, Klaiceva 16, Zagreb, 10 000 Croatia; EUROCAT Central Registry, University of Ulster, Jordanstown Campus, Room 12 L09 Shore Road, Newtownabbey, Co. Antrim, Northern Ireland, BT37 0QB UK; Pediatric Department, Hospital Lillebaelt, Skovvangen 2-6, Kolding, DK 6000 Denmark; Wessex Clinical Genetics Service, Princess Anne Hospital, Coxford Road, Southampton, SO16 5YA UK; Registro IMER, Azienda Ospedaliero-Universitaria di Ferrara, Corso Giovecca, Ferrara, 202 44121 Italy; Division of Medical Genetics, CHUV, Lausanne, Switzerland; Registro Anomalias Congenitas CAPV. Dirección de Salud Pública. Departamento de Sanidad. Instituto BIO-Donostia, Basque Government CIBER Epidemiología y Salud Pública - CIBERESP, Donostia-San Sebastian 1, Vitoria Gasteiz, 01010 Spain; Department of Genetics, University of Groningen, University Medical Center Groningen, Hanzeplein 1, Groningen, 9700 RB The Netherlands; Departamento de Epidemiologia, Instituto Nacional de Saude Doutor Ricardo Jorge, Av. Padre Cruz, Lisbon, 1649-016 Portugal; Department of Health Sciences, University of Leicester, 22 28 Princess Road West, Leicester, LE1 6TP UK; Malta Congenital Anomalies Registry, Directorate of Health Information and Research, G’mangia Hill, G’mangia PTA 1313 Malta; Styrian Malformation Registry, Medical University of Graz, Auenbruggerplatz 14, Graz, AT 8036 Austria; Paris Registry of Congenital Malformations, INSERM U953, Maternite de Port‐Royal, 53 av de l’Observatoire, Paris, 75014 France; Medical Birth Registry of Norway, University of Bergen, Kalfareien 31, Bergen, N-5018 Norway; Health Information Unit, Health Service Executive, Dr Stevens Hospital, Dublin 8, Dublin, Ireland; Provinciaal Instituut voor Hygiene, Kronenbrgstraat 45, Antwerp, B-2000 Belgium; CNR Institute of Clinical Physiology, Via Moruzzi 1, Pisa, I 56124 Italy; Universitätskinderklinik Mainz, Langenbeckstrasse 1, Postfach 3960, Mainz, D 55101 Germany; Institute of Health & Society, Newcastle University, Newcastle upon Tyne, NE2 4AA UK; Malformation Monitoring Centre Saxony-Anhalt, Medical Faculty Otto-von-Guericke University, Leipzigerstrasse, Haus 39, Magdeburg, D 39120 Germany; National Perinatal Epidemiology Unit, University of Oxford, Old Road, Headington, Oxford OX3 7LF UK; Congenital Anomaly Register & Info Service Public Health Level 3 West Wing, Singleton Hospital, Sketty Lane, Swansea, Wales SA2 8QA UK; Institut de Pathologie et de Génétique, Avenue Georges Lemaître 25, Charleroi (Gosselies), 6041 Belgium

**Keywords:** Holt Oram syndrome, Congenital anomalies, Prenatal diagnosis, Epidemiology, Europe

## Abstract

**Background:**

Holt-Oram syndrome (HOS) is an autosomal dominant disorder characterised by upper limb anomalies and congenital heart defects. We present epidemiological and clinical aspects of HOS patients using data from EUROCAT (European Surveillance of Congenital Anomalies) registries.

**Methods:**

The study was based on data collected during 1990–2011 by 34 registries. The registries are population-based and use multiple sources of information to collect data on all types of birth using standardized definitions, methodology and coding. Diagnostic criteria for inclusion in the study were the presence of radial ray abnormalities and congenital heart disease (CHD), or the presence of either radial ray anomaly or CHD, with family history of HOS.

**Results:**

A total of 73 cases of HOS were identified, including 11 (15.1%) TOPFA and 62 (84.9%) LB. Out of 73 HOS cases, 30.8% (20/65) were suspected prenatally, 55.4% (36/65) at birth, 10.7% (7/65) in the first week of life, and 3.1% (2/65) in the first year of life. The prenatal detection rate was 39.2% (20/51), with no significant change over the study period. In 55% (11/20) of prenatally detected cases, parents decided to terminate pregnancy. Thumb anomalies were reported in all cases. Agenesis/hypoplasia of radius was present in 49.2% (30/61), ulnar aplasia/hypoplasia in 24.6% (15/61) and humerus hypoplasia/phocomelia in 42.6% (26/61) of patients. Congenital heart defects (CHD) were recorded in 78.7% (48/61) of patients. Isolated septal defects were present in 54.2 (26/48), while 25% (12/48) of patients had complex/severe CHD. The mean prevalence of HOS diagnosed prenatally or in the early years of life in European registries was 0.7 *per* 100,000 births or 1:135,615 births.

**Conclusions:**

HOS is a rare genetic condition showing regional variation in its prevalence. It is often missed prenatally, in spite of the existence of major structural anomalies. When discovered, parents in 45% (9/20) of cases opt for the continuation of pregnancy. Although a quarter of patients have severe CHD, the overall first week survival is very good, which is important information for counselling purposes.

## Background

Holt-Oram syndrome (HOS, OMIM 142900) is a rare autosomal dominant multiple malformation syndrome characterised by high penetrance and variable expression of upper limb abnormalities, congenital heart defects (CHD) and/or conduction abnormalities [1,2].

Sequence variants of *TBX5* gene, a member of the T-box family of transcription factors, have been identified to affect function in 75% of HOS cases [3–5]. Most are truncating alterations that result in haploinsufficiency, but occasionally sequence variations can lead to extension of the TBX5 protein [6,7]. Different types of sequence variants can cause complete loss or reduction of TBX5 protein function by affecting nuclear localisation of the protein or by disrupting its interaction with other transcriptional cofactors and downstream target genes. Some sequence variants may result in the gain-of-function causing a similar phenotype [8–13]. Since under- and overexpression cause the same phenotype, *TBX5* function is considered to be gene dosage sensitive [13].

The carriers of the *TBX5* allelic variants affecting function show high intra- and interfamilial variability of clinical presentation. These variations can be due to the type and location of the sequence alteration, but also to other modifier factors, e.g., sequence variations in enhancers regulating *TBX5* expression during heart development [14].

Clinical diagnostic criteria of HOS include pre-axial radial ray malformations in at least one upper limb and CHD and/or conduction defects [15,16]. When a heart anomaly is not present, there should be a family history of HOS consistent with an autosomal dominant type of inheritance [2,5,15]. The spectrum of skeletal upper limb defects ranges from an abnormal carpal bone or triphalangeal/fingerlike thumb to bilateral phocomelia. CHD is present in 75% of individuals with HOS. Ostium secundum atrial septal defect (ASD) and ventricular septal defect (VSD) are the most common, but other more severe heart anomalies have been reported as well [2,17]. Conduction heart disease may occur in the absence of structural anomaly. No correlation between the severity of heart and limb defects has been established.

Several large clinical series of HOS patients have been published examining the clinical presentation, differential diagnosis and diagnostic criteria [16,18,19], but population-based epidemiological studies are rare because of the need for large populations and standardised data collection. The prevalence of HOS is estimated to be 0.95/100,000 births, based on a single epidemiological study from Hungary [20]. Most clinical reports refer to live born (LB), children or adults, while there is little information on fetal deaths (FD), terminations of pregnancy after prenatal ultrasound detection of severe anomaly/anomalies (TOPFA) and on patients diagnosed in the neonatal period.

The aim of this study was to investigate the epidemiological and clinical aspects of HOS patients diagnosed prenatally or in the early years of life, using data notified to the European Surveillance of Congenital Anomalies (EUROCAT) database.

## Methods

The study was based on data routinely collected in the period from 1990 to 2011 by 34 EUROCAT registries in 16 countries covering approximately 30% of Europe's annual birth population and extracted from the central database in June 2013. The EUROCAT registries are population-based and use multiple sources of information to collect data on all major structural congenital anomalies, chromosomal abnormalities, and other genetic and environmental conditions presenting with structural defects among LB, FD with gestational age (GA) ≥20 weeks, and TOPFA using standardized definitions and coding. Six registries include cases diagnosed up to one week after birth, three up to one month, and 25 registries include cases diagnosed up to at least one year of age. A detailed description of registries, methods of case ascertainment, data collection and processing is available elsewhere [21,22].

The central EUROCAT database was searched for the International Classification of Diseases (ICD)/British Paediatric Association (BPA) version 9 (759.842, 759.84), ICD10/BPA (Q87.20) and OMIM (142900) codes assigned to cases of HOS. Diagnostic criteria for inclusion in the study were the presence of radial ray abnormalities and CHD, or the presence of either radial ray anomaly or CHD, with family history of HOS [2,4]. Minor anomalies, including those detectable exclusively by x-ray (e.g., carpal bone defects, hypoplasia of hand/arm/shoulder muscles, sloping shoulders), functional limb defects (e.g., minor limitation of movements of thumbs, elbows, or shoulders) or heart defects (e.g., conduction abnormalities) were not systematically recorded, as the EUROCAT focus is on collecting data on major structural anomalies [21].

In two-thirds of EUROCAT registries, clinical geneticists take part in the examination and diagnosing of all patients with dysmorphic features and congenital anomalies, and in the remaining registries clinical geneticists participate in the examination of selected cases [23]. This genetic expertise allows EUROCAT to collect and analyse data on rare genetic syndromes that manifest a characteristic pattern of anomalies prenatally, at birth or in early infancy [23–25]. A medical geneticist reviewed all records including textual description in order to include all relevant clinical information. Local registries were contacted to provide additional/missing information.

The variables included in the analysis were the time of diagnosis (pre- or postnatal), birth outcome (proportion of LB, FD at GA ≥20 weeks, TOPFA), type of congenital anomalies (ICD9/ ICD10 code and written text), infant sex, survival up to 1 week of age, maternal and paternal age at delivery, birth weight (BW), GA at birth or termination of pregnancy, use of assisted reproductive techniques (ART), multiple pregnancy (twin or triplet), and family history. Until very recently, data on genetic testing were not systematically collected and therefore were not included in this study.

### Statistics

Descriptive data are presented as numbers and percentages for categorical data. Means and 95% confidence intervals, based on Poisson distribution, were used to calculate prevalence and a Generalized Linear/Nonlinear model based on Poisson distribution was used for statistical testing of time trends. A difference between two proportions test was used to test prevalence differences between registries and the total prevalence rate. A chi-square test was performed to determine differences in maternal age distribution between HOS and other EUROCAT cases. Maternal age comparisons were restricted to the registries/time period with available maternal age denominator data (63% of EUROCAT total birth population). Statistical analysis was performed using STATISTICA 6.1 StatSoft Inc. 1983–2003 (serial number AGA304B211928E61).

## Results

Between January 1990 and December 2011, a total of 73 cases of HOS were identified in the European population covered by 34 European registries included in the EUROCAT network. There were 11 (15.1%) TOPFA, no FD, and 62 (84.9%) LB. Two of the 62 LB children died in the neonatal period (3.2%). Among the patients for which the time of diagnosis was known (n = 65), 30.8% were diagnosed prenatally, 55.4% at birth, 10.7% in the first week of life, and 3.1% in the first year of life.

The prenatal detection rate was 39.2% (20/51) with no significant change between the 1990–2000 and 2001–2011 periods (*P* > 0.05) (Table 1). The mean GA at prenatal diagnosis by obstetric ultrasound was 18.6 ± 4.6 (range 14–26) gestational weeks. In 55% (11/20) of cases with prenatally diagnosed severe anomaly/anomalies, parents decided to terminate the pregnancy. The mean GA at termination was 21.1 ± 1.8 (range 14–35) gestational weeks. The diagnosis was verified by *post mortem* examination in 10 of 13 cases (eight TOPFA and two neonatal deaths). In 31 out of 51 cases with available data (60.8%), prenatal ultrasound was performed but did not detect any anomaly, although major anomalies (e.g., absence of forearm, bilateral radial aplasia, tetralogy of Fallot, pentalogy of Fallot, etc.) were present in 10/31 patients detected postnatally. Three undetected cases were familial.

**Table 1:** **Outcome of pregnancies and prenatal detection of Holt Oram syndrome in the EUROCAT registries, 1990-2011**

***Monitored period***	***Total no. of patients***	***LB***	***FD***	***TOPFA***	***Prenatally detected/number of patients with available data***	***Prenatal detection rate% (95%*** ***CI)***
**1990-2000**	42	38	0	4	11/28	39.3 (95% CI 21.2-57.4)
**2001-2011**	31	24	0	7	9/23	39.1 (95% CI 19.2-59.1)
**1990-2011**	73	62	0	11	20/51	39.2 (95% CI 25.8-52.6)

*Abbreviations*: *LB* live born, *FD* fetal deaths, *TOPFA* termination of pregnancy for fetal anomaly, *95% CI* 95% Confidence Interval.

Characteristics of the HOS patients are shown in Table 2. The male-to-female ratio was 1.1:1. The mean GA in LB was 39.3 ± 3.1 (range 33–42) in males and 38.4 ± 2.9 (range 27–42) in females. The mean BW was 2998 ± 370 g (range 2240–3800 g) for males and 3241 ± 458 g (range 2400–4200 g) for females. Of 62 live born cases, 96.8% survived the first week of life.

**Table 2:** **Descriptive epidemiological data on patients with Holt Oram syndrome, EUROCAT registries, 1990-2011**

***Characteristics***	***Number of HOS patients N = 73 (%)***	
**Sex**		
Male	38 (52.8)	
Female	34 (47.2)	
Unknown	1	
**Gestational age, wk**	N = 59	
	(live births with known gestational age and sex)	
	M	F
	n = 27	n = 32
<37	5 (18.5)	4 (12.5)
37-41	20 (74.1)	25 (78.1)
≥42	2 (7.4)	3 (9.4)
**Birth weight (g)**	N = 47	
	(live births with known gestational weight and sex)	
	M	F
	N = 20	N = 27
<1500	0	0
1500-1999	3 (15.0)	1 (3.7)
2000-2499	6 (30.0)	8 (29.6)
2500-2999	10 (50.0)	10 (37.1)
3000-3499	1 (5.0)	7 (25.9)
>3500	0	1 (3.7)
**Positive family history**	15.1% (11/73)	
**Mean maternal age (range)**	28.3 ± 4.6 (17–41)	
**Mean paternal age (range)**	32.7 ± 1.3 (19–62)	

wk: weeks.

g: grams.

Eleven families had more than one affected member. Maternal age distribution did not differ significantly from that of the total EUROCAT population (*P* = 0.06). Multiple pregnancies were not noted. Three of 34 patients with known information on ART were conceived by induced ovulation. There were no cases of *in vitro* fertilization (IVF) or intracytoplasmic sperm injection (ICSI). Data on karyotyping were available for 46.1% of cases and all results were normal.

Description of the type and frequency of major congenital anomalies was available for 61 HOS patients. The results are presented in Table 3, together with those on the three large series of HOS patients published so far. Thumb anomalies were present in all patients. Agenesis/hypoplasia of radius was present in 49.2% (30/61), ulnar aplasia/hypoplasia in 24.6% (15/61), and humerus hypoplasia/phocomelia in 26/61 (42.6%) patients.

**Table 3:** **Major congenital anomalies in Holt Oram syndrome: EUROCAT data and in previously published studies**

***TYPE OF ANOMALY***		***EUROCAT STUDY***	***Newbury-Ecob et al., 1996 [*** 16 ***]***	***Hurst et al., 1991 [*** 19 ***]***	***Smith et al., 1979 [*** 18 ***]***
		***N = 61 (%)***	***N = 55 (%)***	***N = 43 (%)***	***N = 39 (%)***
Congenital heart anomalies*					
Atrial septal defect		28 (45.9)	20 (36.4)	20 (46.5)	14 (35.9)
Ventricular septal defect		23 (37.7)	15 (27.2)	3 (7.0)	9 (23.1)
Atrioventricular septal defect		3 (4.9)			1 (2.6)
Pulmonary atresia/stenosis		2 (3.3)	1 (1.8)		1 (2.6)
Double outlet right ventricle		1 (1.6)			1 (2.6)
Aortic valve insufficiency		1 (1.6)			1 (2.6)
Aortic valve stenosis			2 (3.6)		
TrIcuspid atresia		2 (3.3)			
Mitral valve abnormality			3 (5.5)	1 (2.3)	2 (5.1)
Patent ductus arteriosus		3 (4.9)	1 (1.8)	1 (2.3)	1 (2.6)
Pentalogy of Fallot		1 (1.6)			
Tetralogy of Fallot		1 (1.6)	2 (3.6)		
Common arterial truncus		1 (1.6)			
Dextrocardia		1 (1.6)	1 (1.8)	1 (2.3)	1 (2.6)
Right aortic arch					1 (2.6)
Congenital heart disease, NOS		1 (1.6)			4 (10.3)
**LIMBS**					
Thumbs					
	Triphalangeal/digital thumb	24 (39.3)	10 (18.2)	10 (23.3)	24 (61.5)
	Hypoplasia of thumb	16 (26.2)	21 (38.2)	9 (20.9)	13 (33.3)
	Absence of thumb	30 (49.1)	27 (49.1)	10 (23.3)	16 (41.0)
	Accessory/bifid thumb	4 (6.6)			1 (2.6)
	Syndactyly of thumb	3 (4.9)	7 (12.7)		6 (15.4)
Fingers					
	Aplasia of hand and/or fingers	8 (13.1)	6 (10.9)		8 (20.5)
	Hypoplasia of hand and/or fingers	8 (13.1)	7 (12.7)		8 (20.5)
	Syndactyly	20 (32.8)	6 (10.9)		16 (41.0)
Lower arm					
	Agenesis radius	14 (23.0)	13 (23.6)		8 (20.5)
	Hypoplastic radii, short forearms	16 (26.2)	24 (43.6)	9 (20.9)	12 (30.8)
	Ulnar hypoplasia	11 (18.0)	20 (36.4)		7 (17.9)
	Ulnar aplasia	4 (6.6)	4 (7.3)		1 (2.6)
	Synostosis of radius and ulna	12 (19.7)	32 (58.1)		5 (12.8)
Upper arm					
	Humerus hypoplasia	24 (39.3)	28 (50.9)		10 (25.6)
	Humerus aplasia/Phocomelia	2 (3.3)	1 (1.8)	3 (7.0)	1 (2.6)
	Clavicles, abnormalities	8 (13.1)	40 (72.2)		11 (28.2)
**THORAX**					
	Rib anomalies	2 (3.3)			5 (12.8)
**SPINE**					
	Hemivertebra	1 (1.6)			1 (2.6)
	Fusion of vertebrae				1 (2.6)

*Congenital heart anomalies are presented *per* type of anomaly and not *per* patient.

NOS = non specified.

CHD was recorded in 78.7% (48/61) of patients. Isolated septal defects were present in 54.2%, ASD + VSD in 8.3% and various other CHD in 12.5% of cases (Table 4). There were 25% of patients with complex/severe heart defects: tetralogy of Fallot (1), pentalogy of Fallot (1), pulmonary valve atresia (1), atrial septal defect and tricuspid atresia (1), VSD and tricuspid atresia (1), AVSD with multiple ventricular septal defects (2), AVSD with multiple ASD (1), common arterial truncus (1), pulmonary valve atresia (1), aortal valve insufficiency ASD and VSD (1), and double outlet right ventricle (DORV) (1).

**Table 4:** **Type of congenital heart defect in sporadic and familial patients with Holt Oram syndrome (N = 48)**

**Type of CHD**	**Sporadic**	**Familial**	**TOTAL**
**ASD**	13 (34.2%)	5 (50.0%)	18 (37.5%)
**VSD**	7 (18.4%)	1 (10.0%)	8 (16.7%)
**ASD + VSD**	2 (5.3%)	2 (20.0%)	4 (8.3%)
**Complex**	12 (31.6%)	-	12 (25%)
**Other**	4 (10.5%)	2 (20.0%)	6 (12.5%)
**TOTAL**	**38**	**10**	48/61* (78.7% **)**

*Number of HOS patients with available data on type and frequency of major congenital anomalies.

CHD - congenital heart defect, ASD - atrial septal defect, VSD - ventricular septal defect.

CHD was present in all but one familial case (90%). In this maternal case, there was aplasia of the thumb and radial hypoplasia without CHD. Distribution of CHD types among familial and sporadic cases is shown in Table 4.

The prevalence of HOS was restricted to data from 16 registries with an above average prevalence of genetic syndromes and microdeletions (EUROCAT average is 4.8 *per* 10,000 births) according to the Data Quality Indicators developed by EUROCAT (http://www.eurocat-network.eu/content/DQI-2013.pdf). This was done in order to ensure a more homogeneous ascertainment of cases. During the 1990–2011 period, a total population of 5,017,781 births were monitored in these registries and 37 cases of HOS identified. Therefore, the mean prevalence of HOS diagnosed in the early years of life was 0.7 *per* 100,000 births or 1: 135,615 births. The prevalence rates for 1990–2000 and 2001–2011 were 1.1 and 0.4 *per* 100,000 births, respectively (*P* = 0.03) (Table 5). The number of HOS patients and prevalence rates *per* registry are shown in Table 6.

**Table 5:** **Prevalence of Holt Oram syndrome patients in 16 selected EUROCAT registries, 1990-2011**

***Monitored period***	***Total births***	***Total no. of patients***	***Birth prevalence per100 000 (95%*** ***CI)***
1990-2000	2 387 019	26	1.1 (0.69-1.51)
2001-2011	2 630 762	11	0.4 (0.2-0.6)
1990-2011	5 017 781	37	0.7 (0.32-1.08)

*Abbreviations*: 95% CI, 95% Confidence Interval.

Note: the registries were selected according to the http://www.eurocat-network.eu/content/DQI-2013.pdf.

**Table 6:** **Prevalence of Holt Oram syndrome patients per 16 selected EUROCAT registries, 1990–2011**

***REGISTRY***	***Population***	***No. of HOS patients (confirmed familial cases)***	***Prevalence per 100000***
**Styria (Austria)**	229506	2 (2)	0.9
**Antwerp (Belgium)**	341573	2	0.6
**Hainaut (Belgium)**	277204	4 (2)	1.4
**Dublin (Ireland)**	489614	1	0.2
**Odense (Denmark)**	121532	1	0.8
**Paris (France)**	703650	3	0.4
**Mainz (Germany)**	75496	1	1.3
**Cork And Kerry (Ireland)**	141421	1	0.7
**N Netherlands (Nl)**	421026	7 (2)	1.7*
**Vaud (Switzerland)**	166950	4 (1)	2.4*
**Glasgow (UK)**	122803	1	0.8
**N W Thames (UK)**	661527	2	0.3
**Wessex (UK)**	492559	5	1.0
**Thames Valley (UK)**	291759	1 (1)	0.3
**Northern England (UK)**	382900	1	0.3
**Malta**	98261	1 (1)	1.0
**TOTAL**	**5017781**	**37**	**0.7**

*Registries with statistically significantly higher prevalence rates compared to the total average prevalence rate (*P* < 0.05).

## Discussion

The Holt-Oram syndrome is an autosomal dominant condition associated with defective development of the radial ray and cardiac structures, resulting in a wide spectrum of phenotypes. It needs to be emphasized that there is no intellectual impairment which is important information for genetic counselling of affected families. The diagnosis is based on established clinical criteria and can be confirmed by molecular genetic testing in a proportion of cases. The diagnosis is not difficult in the presence of typical clinical features and positive family history. In isolated cases, the differential diagnosis will include hand-heart syndromes type II (Tabatznik) and III (Spanish type), other genetic syndromes with upper limb anomalies (e.g., thrombocytopenia-absent radius syndrome, Fanconi anaemia, *SALL4-*related disorders, ulnar mammary, Kaufmann McKusick, Roberts or Nager syndromes), chromosomal anomalies, associations such as VACTERL and rare teratogenic embryopathies (thalidomide, valproate) [2,26]. In most cases, these conditions can be excluded without major difficulties by careful clinical examination and appropriate diagnostic evaluation including cytogenetic and molecular testing. These tests, including genetic testing for *TBX5*, 22q11.2 microdeletion and Fanconi anaemia, were also performed in the studied patients as a part of the routine clinical evaluation for establishing the final diagnosis. We are not able to present these data, as we have only recently started to collect data on genetic testing in a systematic way.

### Prenatal diagnosis

As HOS is rare and most cases are sporadic, it is mostly reported in familial cases when a more detailed ultrasound examination is performed rather than by prenatal screening [27,28]. The radius and ulna are easy to see at 13–16 weeks, and most cardiac anomalies, with exception of ASD and small VSD, are clearly visible on ultrasound screening for anomalies at 18–20 weeks. Our study showed that over 60% of cases were not suspected prenatally, although many presented with major anomalies that could have been easily detected by prenatal ultrasound and although some cases were familial. In addition, the prenatal detection rate did not improve over time.

Parents of the 9 of 20 patients diagnosed prenatally decided to continue the pregnancy. Two cases were diagnosed late (at 32 and 36 gestational weeks). Among the remaining seven, there were three familial cases suggesting that families accept and tolerate well the clinical consequences of the genetic disorder in their family, as there is no intellectual impairment.

### Clinical manifestations

The distribution of gender was equal. The intrauterine growth and development was not affected. Over two-thirds of HOS patients were born at term and of 50 patients born after 37 weeks of gestation, only four (8%) weighed less than 2,500 g. Likewise, the first week survival was excellent, taking into account a high rate of severe congenital heart anomalies.

By definition, all HOS patients have upper extremity anomalies. Bone abnormalities are usually bilateral and asymmetric, with left side often more affected than the right side. Most skeletal manifestations are visible on inspection but some patients have only functional abnormalities or carpal/metacarpal bone anomalies visible on x-ray. These functional and subclinical skeletal manifestations of HOS were not systematically reported in our dataset, as EUROCAT records major anomalies, while recording of minor anomalies and functional symptoms is optional and would be included in the database only if associated with a major defect. In addition, some of these manifestations are difficult to assess prenatally, at *post mortem*, or in the neonatal period/infancy, when most of our patients were diagnosed.

The most common thumb anomalies were absence of thumbs, followed by triphalangeal/digital thumbs and thumb hypoplasia. Radial hypoplasia was only slightly more common than radial agenesis. The involvement of the ulna was less frequent than of the radius. Upper arm involvement was present in over 40% of patients. These findings are largely in agreement with previous clinical reports [16,18,19]. Due to the previously mentioned methodology issues, synostosis of radius and ulna and subtle shoulder anomalies (narrow shoulders, short clavicles/hypoplasia of head of humerus, and hypoplastic musculature of the shoulder girdle) are probably underreported. Contrary to the observation of Newbury Ecob et al. [16], no difference between isolated and familial cases in the severity of thumb aplasia, radial and ulnar involvement was observed. The predominant ulnar involvement and atypical findings in isolated cases were not noted. Only 1 in 30 patients had phocomelia, which is somewhat less than previously observed [16,29]. Smith et al. [18] found phocomelia to be present more often in familial cases and in females. Both cases of phocomelia found in our series were recorded in males and were sporadic. Some studies noted that skeletal defects were more severe in females than in males [18,29–31], but that males had a greater number of bones involved than females [18]. In this study, we did not observe any sex difference in the type and number of skeletal defects.

CHD occur in approximately 75% of HOS patients. A variety of structural heart anomalies are seen, with ASD and VSD being the most common. The proportion of CHD found in this study was 78.7%. In particular, we observed a higher rate of VSD and a higher rate of severe CHD than in previous reports [16–19]. Our series of patients included a more severely affected subset of patients compared with the clinical series that describe mostly adult patients coming from affected families. HOS patients in our study were diagnosed mostly at birth or prenatally, and pregnancies with severe CHD that resulted in TOPFA were also included. Additionally, some of the VSDs diagnosed at birth would eventually resolve and will not be recorded later in childhood or in adulthood. Although the number of familial cases in our series is small, it is of note that none of them had complex CHD.

Cardiac conduction abnormalities are commonly found in HOS. They are more likely to occur in those with a structural heart defect, but it is reported that about 40% of cases have conduction abnormalities alone [16]. We were not able to study this manifestation of HOS, as recording of functional abnormalities is optional and reported data are not complete. It is therefore possible that some patients with upper limb anomalies and only conduction heart defects will be diagnosed later in adolescence or adulthood when the symptoms of heart disease develop.

Other associated anomalies have also been described in HOS patients and may include craniofacial, tracheal, pulmonary, vertebral, renal and lower limb anomalies [5,32–36]. These can be incidental findings or may be atypical cases of HOS due to specific sequence variants of the *TBX5* gene [11,36,37]. We observed single cases with cleft uvula, brain cyst, spleen anomaly, pyelon duplex, ectopic kidney and hemivertrebra. Lower limb anomalies were found in 3 patients. The types of anomalies (one bilateral hip dislocation, one lower limb shortening, one club foot) would suggest random association rather than the clinical spectrum of HOS.

Two cases with renal anomalies and a case with hemivertebra were evaluated for the possible diagnosis of VATER, but this was not conclusive. The patient with hemivertebra had ASD type secundum, radial and thumb aplasia on the right hand and phocomelia on the left side, which is more indicative of HOS. Data on molecular tests for cases with associated anomalies were not available.

The proportion of familial cases was 15.1%, which is consistent with the results of the only population study on HOS [20] and contrasts with clinical reports citing that up to 85% of cases are familial due to the ascertainment bias [16,20,23]. The mean parental age did not differ from the general EUROCAT population, although a wide range (19–62 years) of paternal ages was observed.

ART methods are known to be associated with a higher risk of congenital anomalies, especially congenital heart defects [38,39] and are a risk factor for some genetic syndromes [25,40]. In addition, ART could facilitate propagation of pre-existing mutations that are associated with impaired fertility, e.g., in the sperm of older men [41]. Our sample, although small, did not show correlation between the ART techniques and HOS.

### Prevalence

The results of this European study show that HOS is a very rare condition with an average prevalence of 0.7 *per* 100,000 births and a high regional variation [range 0.3 (N W Thames)-2.4 (Vaud) or 1: 330,763 to 1: 41,737]. The prevalence is higher in registries were familial cases are recorded. The mean prevalence of 1: 135,615 births represents a minimum figure and refers to a group of patients with obvious clinical presentation.

The only population-based study on HOS was conducted in Hungary covering the 1975–1988 period [20]. The established prevalence of 0.95 *per* 100,000 births is in agreement with the prevalence of 1.0 *per* 100,000 births recorded in our dataset for 1990–2000. Interestingly, there is a significant decrease in the prevalence rate for 2001–2011 period, for which there is no obvious explanation and requires further monitoring.

### Strengths and limitations of the study

This is the largest population-based study on HOS to date. The results are based on the same methodology and case description and there is sufficient genetic expertise to have confidence in the clinical diagnosis. All cases met strict diagnostic criteria as proposed by McDermott [2005, 2013], but a proportion of mild cases without overt anomalies within the radial ray or those with only conduction heart defects are missed. Another study limitation is that we have data on genetic testing only for a minority of patients, which does not allow genotype phenotype analysis or confirmation of cases with associated anomalies.

## Conclusions

HOS is a rare genetic condition with regional variability. It is often missed prenatally in spite of the presence of major anomalies. When discovered, half of the parents will opt to continue the pregnancy even in the presence of severe limb defects and CHD. Our data indicate that the number of severe CHD in HOS is underestimated since they are present in one quarter of HOS patients. In spite of this, the overall first week survival is good, which is important information for genetic counselling of affected families.

## Abbreviations

HOS: Holt Oram syndrome
EUROCAT: European surveillance of congenital anomalies
LB: Live born
FD: Fetal deaths
SB: Stillborn
BW: Birth weight
GA: Gestational age
TOPFA: Termination of pregnancy for fetal anomaly
CHD: Congenital heart defects
ASD: Atrial septal defect
VSD: Ventricular septal defect
AVSD: Atrioventricular septal defect
ICD: International classification of diseases
BPA: British paediatric association
OMIM: Online mendelian inheritance in man
ART: Assisted reproductive techniques
IVF: *in vitro* fertilization
ICSI: Intracytoplasmic sperm injection
